# ZARATAMAP: Noise Characterization in the Scope of a Smart City through a Low Cost and Mobile Electronic Embedded System

**DOI:** 10.3390/s21051707

**Published:** 2021-03-02

**Authors:** Unai Hernandez-Jayo, Amaia Goñi

**Affiliations:** Deusto Institute of Technology (DeustoTech), University of Deusto, 48007 Bilbao, Spain; amaiagoni@opendeusto.es

**Keywords:** Internet of cities, dynamic noise mapping, machine learning, noise characterization

## Abstract

Like other sources of pollution, noise is considered to be one of the main concerns of citizens, due to its invisibility and the potential harm it can cause. Noise pollution could be considered as one of the biggest quality-of-life concerns for urban residents in big cities, mainly due to the high levels of noise to which they may be exposed. Such levels have proven effects on health, such as: sleep disruption, hypertension, heart disease, and hearing loss. In a scenario where the number of people concentrated in cities is increasing, tools are needed to quantify, monitor, characterize, and quantify noise levels. This paper presents the ZARATAMAP project, which combines machine learning techniques with a geo-sensing application so that the authorities can have as much information as possible, using a low-cost embedded and mobile node, that is easy to deploy, develop, and use.

## 1. Introduction

Nowadays, the impact of noise on people’s health is commonly accepted and well known. However, it is important to differentiate between social or environmental noise and occupational noise. Environmental noises, such as the ones produces by roads, railways, or industrial sources, can be the origin of some health effects, such as annoyance, sleep disturbance, or cardiovascular disease. Meanwhile, occupational noise or industrial noise is the sound level to which workers are exposed [1]. This work deals with the analysis of environmental noise in the framework of a city. The European Environment Agency (EEA) has identified that environmental noise, and in particular road traffic noise, remains a major problem affecting the health and well-being of millions of people in Europe [2,3] and it is also an indicator of sustainable local development [4], being yet another element in the creation of poverty and inequality [5].

As a first step towards taking action against noise, a campaign of measurements is needed to quantify its levels and impact, according with the European Noise Directive [6]. To this end, noise mapping is one of the most informative and useful tools to provide noise monitoring and control in cities [7]. Typically, public administrations carry out periodic measurement campaigns using fixed locations as in Benocci et al. [8], sometimes with a periodicity of years, as it is estimated by municipal regulations. However, nowadays, thanks to the advances in technology, it is possible to carry out measurement campaigns using mobile nodes, which allow to obtain more data in larger locations of the city. A simple example could be the use of users’ mobile terminals as acoustic sensors to collect information in those places where they move [9]. However, the success of these solutions depends largely on citizens’ interest in participating. Rapid evolution of electronics and communications has given rise to wireless solutions that make numerous measurement and data collection points available in cities. Thus, wireless sensor networks (WSN) are a technological alternative that make it possible to increase the number of measuring stations in the city [10], in a simple way that does not require large economic investments.

In any case, this type of solution aims to increase the number of measurement points in the city, to carry out measurements over a longer period of time, and to obtain contextualized information on noise. A combined alternative in the proposal in the ZARATAMAP project, where the aim is to involve public administrations to offer the public electrical bicycle-sharing service as mobile nodes.

During the conceptualization of the project, different means of transport were proposed in which to board the prototype to be developed in the project: public buses, taxis, or electric bicycles. Following the requirements’ analysis carried out at the beginning of the project, it was decided to use e-bikes as the mobile node. The choice was based on the low or non-existent noise level generated by the bicycle itself. In addition, after analyzing different techniques that can be used to eliminate the background noise caused by motor vehicles [11,12,13], it was found that the high computing power needed to carry out these signal processing tasks required the use of more powerful but more expensive embedded systems, which made the project unfeasible.

The project proposes the installation of nodes equipped with the capacity to sensor noise, geo-locate it, and transmit the captured information to a central service where it can be monitored, processed, and provided with operational support services. Among the services to be provided, it is the characterization of the possible main sources of noise in the city. Noise analysis is also an area of research in which machine learning has been shown to have a wide range of applications, being one of them, the prediction of traffic volume as a function of vehicle generated noise as in Alam et al. [14] or to determine noise prediction in industrial scenarios [15]. From our point of view, we can apply this knowledge in the field of smart cities to determine the main sources of noise at specific points in the city and thus provide more detailed information to public administrations in order to take the best measures to help mitigate that source of noise. The final solution provides a module in charge of automatically classifying the main sources of noise in each of the monitored points of the city. In this framework, the aim of this article was to show the developments that are being carried out within the scope of the ZARATAMAP project. Thus, the article presents the tasks that have been carried out so far, including a description of the prototype of the integrated mobile node, as well as the information management centre and the machine learning module developed as an aid to the exploitation and identification of noise sources in the city.

This paper is structured as follows: Section 2 introduces briefly the state-of-the-art of acoustic urban sensing projects. Section 3 explains the design of the low-cost mobile acoustic sensor proposed, while Section 4 describes the architecture of ZARATAMAP solution. Section 5 shows the results obtained so far, and finally, conclusions and future work area presented.

## 2. Related Work

Traditionally, urban noise measurements in a public administration framework have been performed by licensed professionals, who capture and analyze data using certified instruments or sensors [16]. The recordings are executed at established times of the day in a predefined set of locations, due to the cost the process of moving the equipment entails. In this context, the noise maps generated from those measurements, produced, typically, on a 5-year basis, are representative of fixed urban space- and time-frames, not enough for a real city-wide picture. However, although it is a valid methodology, it does not reflect the acoustic reality of the city by not contemplating, for example, measurements over long periods of time or in different and random locations. Once the captures have been made, it is possible to make estimates of the acoustic impact using specific software and mathematical approaches [17].

With an increasing importance of noise pollution in urban areas, in the last decades the demand for more representative and reliable data has undergone a great expansion, requesting the consolidation of data captured in a 24-h span in the entirety of the concerned geographical locations. The usual approach to Real Time Noise Mapping consists of implementing a localized Noise Monitoring Network that collects noise data continuously and transmits them to a data center in which a noise model software is running. The role of the noise model software is to re-scale some pre-computed partial noise maps according to the measured data (each noise monitoring station should refer to each relevant single noise source as road, railway, etc.), and to sum the new re-scaled partial maps together, in order to obtain the whole area noise map to be published and continuously updated on a web site [18]. Then, in order to achieve 24-h noise monitoring, we study the available technologies from two perspectives: the use of static sensors networks and mobile noise monitoring using ubiquitous nodes.

### 2.1. Static Sensor Network Based Noise Monitoring

Designed for specific applications, there have been several proposals for static sensor network-based noise monitoring schemes in the last years. One of the engineering fields around which these solutions have been developed has been the Wireless Acoustic Sensor Networks (WASN) technology [19], based on the deployment of a network of small, low-cost, smart sensor nodes. This methodology is used in different scenarios as underwater [20] and environmental [21] circumstances.

An example of this approach can be found at the work of Dong et al. in  [22], where a traffic noise monitoring system is presented in the Chinese city of Xiamen. A similar approach is used by Kazmi et al. in [23], where a static IoT-based platform based on multifarious sensing devices is developed to monitor and manage road traffic noise in Galway (Ireland). A similar solution is developed by Masques et al. in [24], where it is proposed a cyber–physical system for data collection and web software for data visualization about noise impact in Residential Buildings using open-source technologies. Noise is not only a problem in open spaces. The impact of noise in indoor spaces is measured and studied by Son et al. in [25], where sound pressure level (SPL) is measured using an integrated system that deploys an microphone sensor-based array also to determine the noise source localization, calculating the estimated time difference of arrival.

All these developments are based on smart and low cost platforms. Once the sound samples are recorded, this information must be visualized on a map to provide information on the acoustic impact in a geo-tracked manner. Vogiatzis et al. in [26] provide a good overview about different tools to handle a large amount of data related to environmental noise and the urban soundscape. At [27], Bocher et al present an open source implementation of a noise mapping tool fully implemented in a Geographic Information System compliant with the Open Geospatial Consortium standards.

Nevertheless, these approaches becomes difficult to scale up when more geographical or temporal measures are required. Moreover, noise maps generated from static measurements, use to consider assumptions in the predictive models, which could imply the loss of key features of environmental noise, such as the characterization of its temporal evolution [28]. These drawbacks can be overcome with the use of mobile wireless sensor networks.

### 2.2. Mobile Noise Monitoring on Ubiquitous Nodes

An alternative to the static sensor grid explored in the previous examples focuses on the placement of the WSN in moving platforms, allowing the noise sensing from an indefinite set of locations. In that regard, one of the first examples that can be found in this research area is [29], where a Mobile Sensing Unit (MSU), linked to a set of GPS coordinates, was developed. In detail, this project tested a network of devices placed in cars and buses of Seoul’s public transport system, measuring temperature, humidity, and noise levels (no additional signal analysis was performed). More recent work, such as the one developed by Dekoninck et al. [30], proposed, like the work presented in this paper, the use of bicycles as mobile nodes, for measuring traffic, noise, and air pollution. In this work, a professional sonometer manufactured by Svantek is adopted to carry out the measurements. This approach provides great precision in the measurements, but on the other hand it increases considerably the price of the solution and does not make possible its massive deployment in a fleet of bicycles, as it is proposed to achieve with the ZARATAMAP project.

Another different approach is the one cited by Pokorny et al. in [31]. The scope of this work is the automatic recognition of a cyclist’s route between fixed endpoints, the route direction, and the route progress, using the audio data recorded with a cell-phone attached to the cyclist. It could be another example of geo-sensing, but in this case, its final application is not devoted to noise mapping.

Instead of using vehicles, NoiseSense project [32] is based on mobile crowdsourcing over smartphones, where noise levels are measured in the surroundings of the user. Thus, taking advantage of this information city’s noise pollution footprints are generated. NoiseProbe [33] proposes a similar scope including a participatory sensing system that makes use of inbuilt microphones in mobile devices for ambient noise pollution monitoring. Both solutions aim to provide each user with information about their personal exposure to noise pollution, but always on the basis of citizen participation, an aspect which is subjective and which requires a high level of participation if sufficient information is to be obtained to produce a dynamic noise map of the city.

Based also in this concept of participatory and crowd-sourced noise mapping, other initiatives have been carried out. For example, Aiello et al. at [34] describe how social media data are used to create an urban sound dictionary and also to track these sounds in the context of the city of London and Barcelona. Another example is the work presented by Guillaume et al. OnoM@p [35], where the system aims at offering a simple mobile-based application to gather noise data recorded by volunteers citizens. It is a similar approach as the one shown by Picaut et al. in [36], where users can use a mobile application (NoiseCapture) to capture and geo-reference the sound around them in a simple way. As a complement, once the measurement is done, a user can introduce a ‘Description’ of the sound and an evaluation of his own perception of the sound environment

### 2.3. Noise Characterization Systems

Both systems based on static sensors and those using mobile nodes, are able to record real noise data. However, they cannot characterize noise sources by themselves, which is vital for the advanced evaluation, mitigation, and control of any noise. Using information provided by an IoT platform, multiple systems and devices can be interconnected, which provides a large amount of data that can be combined in an orderly and strategic way to be used to catalog and characterize the main sources of noise in the city. Machine learning could be a good candidate performing this task since it can be trained to characterize sound noise through analyzing examples under various models and approaches. A good introduction to the application of machine learning in acoustics is described by Bianco et al. in [37].

Examples of research works in this area are [13,38,39]. Another application example is Sounds of New York City (SONYC) [40]. This project is based on a low-cost, intelligent sensing platform capable of continuous, real-time, accurate, source-specific noise monitoring. This platform also provides an urban sound taxonomy, annotated datasets, and various cutting-edge methods for urban sound-source identification.

In order to carry out a classification of noise sources, different techniques can be used. One of these is the use of classifiers based on Support Vector Machines (SVM). These machines can be trained to build a model that predicts the class of a new sample. One example is used by Shaukat et al. in [41], where this technique is used in combination with Convolution Neural Networks (CNN) to recognize different sounds from the daily living activity of humans. Other more advanced techniques may include Local Binary Pattern (LBP), for which the histogram representing the frequency spectrum of the noise is used and processed as an image [42]. Alternatively, Gaussian Mixture Model can be used a probabilistic model for representing normally distributed subpopulations within an overall population, a popular technique used for speech recognition or as Jeon et al. propose in [43] for classifying environmental sounds using drones.

## 3. Mobile Acoustic Sensor Design

### 3.1. Embedded System Platform

As it has been introduced before, one of the goals of this project was to obtain a low cost and fast development platform that could be easily deployed on bike-sharing services. Therefore, it is necessary to analyze the most common embedded systems that provides these functionalities besides of having a set of inputs and outputs that facilitate the development of a first prototype to validate the proposed solution. After the testing phase and any necessary improvements, the company in charge of the prototype industrialization will determine which approach will be taken for the product consolidation. During this prototyping stage, standard and common embedded system have been analyzed, mainly because nowadays their functionalities, characteristics, and the tools that they provided, are quite enough for satisfying the goals of this prototype.

#### 3.1.1. Arduino Uno

Arduino Uno by Arduino©, Somerville, MA, USA is the most used board from the Arduino family, being specially popular between beginners to microcontrollers and programming. It can be connected to a computer via USB cable for development and power supply, that can also be obtained with a 7 to 12 V AC-DC adapter or battery. Regarding peripheral connection, it provides 14 digital and 16 analog input/output pins, all operated by a 16 MHz ceramic resonator.

With an ATmega328P microcontroller by Microchip©, Chandler, AZ, USA as the core basis, it provides a 32 KB Flash Memory, 2 KB SRAM and 1KB EEPROM. Its power consumption is calculated to be 46 mW/MHz or 46.5 mA, at 16 mHz in normal operating conditions, reaching a 34 mA consumption in Deep Sleep mode. Regarding communications, it offers one Serial connection, one SPI, one TWI, one UART, one USB, and one I2C connection. Lastly, it integrates a built-in A/D Converter with a 10 bit resolution and 6 channels. All these characteristics are available in really small size and are cheap (around 24 €).

#### 3.1.2. Raspberry Pi 4

Raspberry Pis by Raspberry Pi Foundation are one of the most popular and versatile boards in IoT applications, being model 4 the most recent one. With a Broadcom VideoCore VI GPU as core, it works at 1.5 GHz, consuming an average of 350 mA and 1.9 W in its nominal conditions. Unlike many boards used for the same purposes, it embodies a considerably big memory solution: 1, 2 or 4 GB of SDRAM, with the possibility of attaching a microSD of any size.

Regarding connectivity, it offers 40 GPIO ports and various advanced communication alternatives, such as USB 2.0 and 3.0, two micro-HDMI 2.0, TVDAC, Gigabit Ethernet, or Bluetooth 5.0. However, it does not provide an embodied A/D converter, demanding an external component for that function. Without taking this attachment into account, this board costs 64 € (4 GB), and has 88 mm × 58 mm × 19.5 mm dimensions.

#### 3.1.3. FRDM-KL25Z

The FRDM-KL25Z manufactured by NXP©, Eindhoven, Netherlands, is an ultra-low-cost development platform based on a ARM Cortex M0+ processor running at 48 MHz. The standard board integrates 128 KB FLASH and 16 KB RAM memories and 66 GPIO ports. The most distinctive features of this device are its built-in sensors, that include an MMA8451Q 3-axis accelerometer by NXP©, Eindhoven, Netherlands and a capacitive touch sensor. Additionally, more analog sensors and actuators can be attached to it though the 16 bit ADC or 12 bit DAC, or via communications like: SPI (2), I2C (2), UART (3), PWM or USB. The market price of the FRDM-KL25Z generally hovers over 20 €, for a final product of 81 mm × 53 mm.

#### 3.1.4. STM32F401RE

Based on an ARM©Cortex M4 32-bit RISC core that can run at frequencies as far as 84 MHz, the STM32F401RE mnufactured byt STMicroelectronics©, Geneva, Switzerland, is a considerably complete option for IoT applications. In detail, it brings a memory protection unit (MPU) for both its 512 KB Flash and 96 KB SRAM memories, which upgrades security in Internet of Things architectures using it.

This development board offers 81 I/O ports, all of them 5V-tolerant and with interrupt capabilities. Furthermore, it places 12 communication interfaces at the user’s disposal: 3 I2C, 3 USART, 4 SPI, USB 2.0, and an SDIO interface. For appended analog devices it provides a 12 bit, 16 channel A/D converter, and a general-purpose DMA. All these features are assembled in a device with a final power consumption of:

- Running: 146 mA/MHz (no peripheral on).
- Stop mode: 42 mA @25oC.
- Deep power down mode: 10 mA @25oC.
- Standby: 2.4 mA @25oC (without RTC).

In addition to these common characteristics, the STM32F401RE provides some singularities, such as 11 timers, RTC hardware-accurate calendar, built-in CRC calculation unit, 96-bit unique ID, DSP instruction integration or a single precision Floating point unit (FPU). All the mentioned functions and elements are gathered in a 82.50 × 70 mm^2^ and 14 € product.

#### 3.1.5. Comparative Analysis

The selected embedded system for the project development has been the STM32F401RE. This decision has been made following the project’s objectives as criteria and after the analysis summarized at Table 1. First of all, two of the core requirements set for the application are to ensure accurate noise acquisition and to prevail a low-price. For this reason, Raspberry Pi 4 is discarded, as the total price is too high and has no built-in ADC, which would be translated into acquiring a specific peripheral for this purpose, alternatively implying a price increase and the risk of losing accuracy in noise measurement.

When it comes to Arduino Uno, the drawbacks that make it unsuitable for this application are its connection limits and clock speed. To develop exhaustive noise maps, it is imperative that peripherals that provide GPS, WiFi, and Microphone functionalities are attached to the microcontroller. Many times, these peripherals require more than one I/O port and are connected via UART protocol. Therefore, having only 14 GPIO ports and one UART connection exceedingly limits the options for these modules. Additionally, for an accurate representation of audio, high sample rates are required, and to perform them, the device’s clock must be fast, which questions if a 16 MHz frequency core is enough.

Ultimately, the decision falls into the FRDM-KL25Z and the STM32F401RE. In this case, the choice is not based on what is lacking, but on what is to offer. Both boards have similar capabilities, but ST’s product offers more characteristics and functionalities for a lower price, making it more adequate for the creation of the autonomous sonometer. Furthermore, the STM32F401RE has the support of a more active developer community which provides examples, libraries, and documentation, making the development process more efficient, rapid, and competitive.

### 3.2. Peripheral Selection

**Figure 1:**
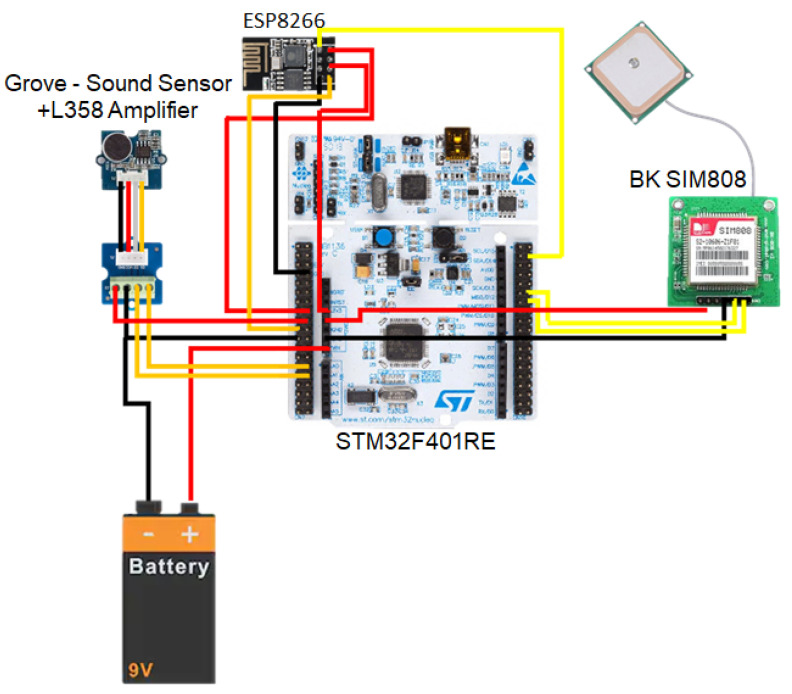
Peripheral block diagram.

Once the hardware computing platform has been selected, it must be equipped with the necessary interfaces and peripherals to be able to collect the ambient sound, geo-locate it, and send it to the central server. To do this, the STM32F401RE platform must deploy an acoustic sensor, a GPS positioning system, and at least one wireless communications interface, as well as an autonomous power supply system, as shown in Figure 1. In the following subsections a brief analysis of different options for each of these elements is made, and the option selected for each of them is justified.

#### 3.2.1. Acoustic Sensor

A sound sensor is defined as a module that detects sound waves and converts them into electrical signals, as long as the signal frequencies are within the working range of the sensor. As the aim of the work carried out was to analyze the acoustic impact on people, it is necessary to arrange a sensor able to work in the frequency range between 20 Hz and 20 kHz. In addition, low frequencies are included into this analysis due to the impact that they can also produce on hearing [44]. In addition, it is recommended to use a low power consumption sensor, while maintaining adequate performance in terms of sensitivity and Signal to Noise (S/N) ratio. Although the IEC 61672 standard [45] identifies two classes of acoustic meters (class 1 and class 2) according to the acceptable error tolerance, this consideration must be considered during the certification and industrialization phases, being out of scope of this stage of the project. It is assumed that standardization work will be necessary later, as well as adjustments in the calibration of the final product. During prototyping phase we want to validate that the general concept of a mobile acoustic sensor is feasible and that it can offer a series of measures and services for the characterization of the acoustic impact.

Considering the frequency range, the only suitable module from Table 2 is Grove Sound Sensor, as the other alternatives, even if they outstand the chosen module in other characteristics, are not capable of acquiring sounds lower than 50 and 100 Hz. Analyzing the Grove Sound Sensor more in detail, its nominal operating voltage is 5 V, with a power supply that can be directly obtained from the STM32F401RE that it will be connected to. This characteristic simplifies also the design of the power supply system.

On the other hand, Grove Sound Sensor is part of the Grove System, a standardized connector system developed to make peripheral connection easier for the developer. To use it, a base unit is required, a set of plug-in adapters that can integrated within the microcontroller board itself or be attached through an external platform to it. In this case, the STM32F401RE does not come with any built-in Grove connectors, but external options are available on the market. However, to reduce costs and size of the prototype, it has been decided not to use a base unit. This is possible because all Grove devices are prepared to be used without the connector, wiring standard cables to the module’s Grove attachments directly or using a Grove Screw terminal, as it is the case.

Nonetheless, even if being part of the Grove kit could seem as an unmeaningful additional task in this case, it provides a huge benefit that reinforces the selection of this peripheral over others: a very-active, tutorial-driven developers’ community. In fact, Grove’s sound sensor is gathered within the seeedstudio online development platform, that even if it is more focused Arduino-based applications, provides many examples and tutorials around the Grove Sound Sensor.

Sound level meters or sonometers are classified into two different classes defined by International Standards such as IEC 61672-1:2013 [45]. The classification into class 1 or class 2 is based on several characteristics and criteria such as accuracy, performance, and calibration. Basically, the main difference between class 1 and class 2 lies in the accuracy and tolerance being class 1 sonometers more precise and class 2 more a general grade meters. In this respect, the chosen microphone must be calibrated together with the processing software developed in the project to establish the degree of compliance with both classifications. This work is currently in progress at this stage of the project so that calibration and adjustment of the final acoustic sensor can be carried out.

#### 3.2.2. WiFi Module

With the popularization of Internet of Things applications and their importance in the 4.0 Industry schema, the use of WiFi modules with microcontrollers is widely spread, which has traduced in a considerable increase in the development of this attachments. In that extent, there are many different options available in the market that offer very similar functionalities and characteristics, as can be observed in Table 3.

In this case, as the characteristics that different modules offer are very similar, the choice criteria has focused in the maturity and price. Since the beginning of WiFi connector devices, Espressif’s ESP-01 module has been the market leader, being the selected option for most embedded applications that require Internet connection. This popularity increased the number of modules produced and sold in a very competitive field, which has ultimately translated in a very low price for the device, finding its offer at prices lower than 1 €. Additionally, it has meant that the number of references in the Internet is very wide, providing examples and already designed applications that are very easy to find and follow. For this reasons, the ESP-01 has been the selected module for this project.

#### 3.2.3. GPS Module

The selection of the GPS Module is slightly more subtle than the one for the previous hardware devices. The three analyzed modules have similar accuracy, sensitivity, and update rate values, and fairly analogous operating voltages and dimensions. If starting times are considered, Grove GPS Module seems like the best option, followed by Ublox NEO-6M by U-Block©, Thalwil, Switzerland and BK-SIM808 by AND Technologies. In contrast, from the price perspective, BK-SIM808 is slightly better than the others (Table 4).

The final criteria for the choice, however, has been neither of those, but the scalability options the modules provide. A paramount feature for this project is to avoid, as much as it is possible, any data loss, that is likely to happen if no reliable WiFi access point is found available. To avoid it, in a future progression of this project, an additional network access could be introduced, for example, through a 4 G connection, provided by an SIM card. If this is to be made, the BK-SIM808 GPS Module provides a built-in slot for an SIM card, that could be used. This way, the additional feature would be implemented without compromising the low price, nor the low power consumption of the sonometer. For this reason, the BK-SIM808 has been considered to be the best option for the project.

### 3.3. Antialiasing Filter

In signal processing, an undesirable effect to be regulated is aliasing, a phenomenon that causes unique signals to turn into indistinct (aliases of one another) during the sampling phase. Original signals affected by aliasing can not be reconstructed from their digital representation in a univocal way, as their digital representation is shared with other input waves. When working with audio signals, it is critical to control the aliasing effect to ensure the wave is obtained, stored, and interpreted in a representative manner. In applications where the sample rate is fixed and known, the usual procedure to avoid aliasing is to include an anti-aliasing filter before the sampler. In detail, it is a low-pass filter designed for a cutoff frequency determined by the Nyquist theorem, that cancels all signals with a frequency higher than this one.

In detail, Nyquist sampling theorem states that an analog signal can be reconstructed through its sampled equivalent, in other words, through samples taken in defined, consistent time intervals. To do so, the theorem proposes the calculation for an optimum sample rate, that is sufficient to perform the transformation from digital to analog signal, without losing critical information in the process. Using this sample rate during the sampling process, that is, sampling at a frequency equal to the Nyquist frequency, disambiguities in Digital-to-Analog conversions can be avoided.

In particular, this sufficient sampling rate (FS) is declared to be any frequency greater than 2B samples per second, being B the maximum frequency the original signal is expected to reach. In audio applications, the usual sampling rate is 48,000 Hz. Therefore, to avoid aliasing, a unity-gain low-pass filter for a Nyquist frequency of 24,000 Hz is implemented.

**Figure 2:**
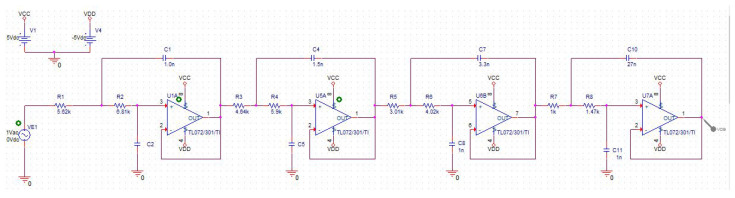
Antialiasing filter design implemented in Orcad Pspice.

There are different topologies that can be applied, depending on the technology foundation of the filter: Cauer (passive), Sallen-Key (active), multiple feedback (active), Biquadratic (active), etc. In case of this project, for the design of the antialiasing filter, an active technology with a Sallen-Key topology has been selected, due to its performance and simplicity. In fact, this topology makes possible the implementation of the filter with a small amount of components, obtaining a dimension-wise small circuit.

for the design of the filter, it has been used the Texas Instruments’ online filter designer tool (https://webench.ti.com/filter-design-tool/filter-type accessed on 1 March 2021) and Orcad PSpice for the simulation and electronic design. In the process, diferent operational amplifiers have been tested and both resistances and capacitors have been adjusted, focusing on the development of a circuit whose elements are standard and easily attainable for a low price. As a result, the final circuit obtained using Orcad Pspice is shown in Figure 2.

### 3.4. Embedded Database

In many IoT applications, just as the ubiquitous sonometer, sensor measurements are paired and stored alongside their time-stamp, a practice that allows a posterior analysis of the data. With the fourth industrial revolution, those necessities are being more and more demanded, which has impulsed the development of databases specially optimized for the management of these data units, called time series. These databases, labeled as time series databases (TSDB), are prepared for supervising time related changes in the measurements, focusing on life-cycle, summarization, and large-range scans of extensive amounts of data.

According to the ranking performed by DB-Engines in April 2020 at the top of TSDBs is InfluxDB, an open source database designed for high-write and query-loads of time series, widely used for IoT applications due to these characteristics. InfluxDB is packaged with a SQL-like query language, called In uxQL, to interact with data, and a powerful API that integrates a variety of functionalities, from HTTP connections to client libraries for languages like Java and Python, that make the development process faster and easier. Regarding its data model, InfluxDB offers a data sending protocol that is different to usual databases, time series or not. Specifically, it provides a line protocol that has the following structure:

measurement-name tag-set field-set timestamp

In this protocol, the measurement-name, that would be comparable to the name of a table in a relational database, is a string; the tag set, a collection of key/value pairs, in the format key = value, where the value is specified to be always a string, and different pairs are separated by commas; the field set, a collection of key/value pairs, in the same format as the tag set, but with values that can be int64, oat64, bool, or string; and the timestamp, a number or string, depending on the format and precision (second, millisecond, microsecond, or nanosecond) chosen. Once data arrive to the database, they are organized in a columnar style format, ordering each field sequentially for blocks of time, which makes later calculations faster and more efficient. Additionally, this ordering system does not constraint the number of tags available for the user, to which there is no limit. All these characteristics make InfluxDB the best option for this project, centered around time based geo-positioned noise measurements.

### 3.5. Firmware Implementation

The software architecture of the project is divided into two different levels. Firstly the firmware developed and deployed in the embedded system is described in the previous sections. Secondly, a complete client-server architecture has been designed and implemented for the transfer of the information from the embedded system (client) to a cloud application (server), which is responsible for storing the information and providing a series of functions for the analysis and characterization of the noise captured by the embedded system. This section describes the architecture and main characteristics of the designed firmware embedded into the STM32F401RE platform.

The main purpose of the implemented firmware is to capture audio samples in specific time blocks, using a sampling frequency of 48,000 Hz, and processes them using A-Weighting and Octave filters, obtaining the RMS values of the signal. These values are stored and geo-localized, obtaining a set of 11 audio samples per GPS position: 10 samples per octaveband and the total noise contribution LeqA. These geo-localized data units are then sent to the operation support server, using the the previously introduced WiFi interface. More precise frequency bands can also be used, such as 1/3 octave filters, but this may require more processing power from the microprocessor used, as well as more memory capacity as the number of samples increases.

**Figure 3:**
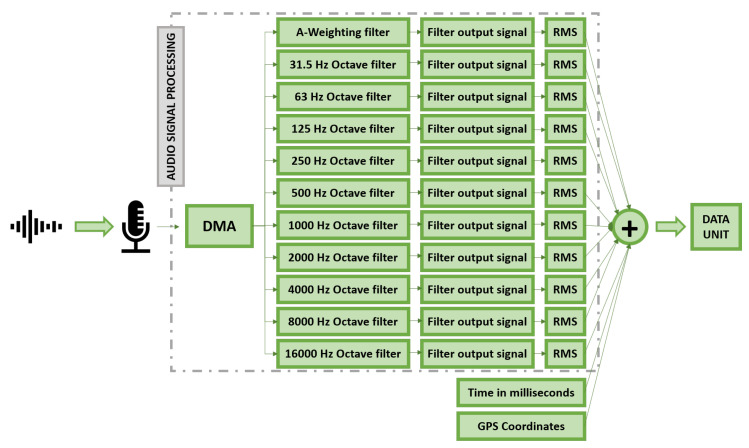
Block diagram of the audio signal processing schema.

To understand the structure of the firmware, it is necessary to introduce the following defined structures, that produce the data unit upon which the application is designed (Figure 3):

- Frame: defined as all the audio lectures made by the microphone at a frequency of 48,000 Hz during a configurable time period. This time period is determined by the size of the frame specified at the config.h file. Then, a frame size of 3000 will mean that each frame stores audio values for an equivalent time of 1/16 s (3000/48,000).
- Data Unit: a data structure created into the project that contains the following field:
  – Noise: it is a float array that stores the RMS values of the original signal, the A-Weighting equivalent, and each of the octave-band analysis, gathered in the setup time period. By default, it relates to data obtained in 1 s (16 frames).
  – Millis: 64-bit number that stores the time in which the data unit was measured, in a time format of milliseconds since 1 January 1970. This value is established at the beginning of the data unit, when the first frame is being processed.
  – Coordinates: Pointer to a structure instance of type Coordinates (structure that contains three float fields, for storing latitude, longitude, and altitude values). This instance stores the geographical positioning determined for the data unit, and is shared by all the data units that are established to have the same GPS positioning.
- outBuffer: defined as the array that contains all of the data units.

In summary, the the operation of the firmware can be described as follows:

1. The device captures noise from the microphone, using Direct Memory Access (DMA).
2. When half of the DMA has been filled, an interruption is generated and signal processing is called.
3. The data frame that has just been obtained is processed using filters, and the end result is added up to the running rms-tracking temporary variable of each of the filters. These variables store the sum of all the values regarding a unique filter, in order to ultimately calculate the RMS of the whole data unit. During this process, audio data are still being obtained and stored in the other half of the DMA buffer.
4. When all data frames that constitute a data unit have been obtained, RMS values of the data unit are calculated and the recollection of the next data frame is started.
5. When as many data units as specified in NUMBER OF SECONDS WITH SAME GPS have been gathered, GPS Positioning sequence is executed. During this sequence, audio data is still being obtained by the DMA, but is not processed or stored in outBuffer. It is like if audio gathering would be hold on pause.
6. When the GPS Positioning finishes, the data units previous to that moment will have been linked to their GPS Positioning, and audio data processing will be resumed.
7. When outBuffer is full or the time specified in SECS BETWEEN WIFI DUMP passes, events that will happen almost simultaneously, WiFi dumping sequence starts (if no GPS Positioning is currently being made, case in which the WiFi sequence will wait until the positioning finishes). During this sequence, same as in the GPS Positioning one, audio processing will be put on pause.
8. When data dumping finishes, audio gathering will resume, and the whole process will start again.

#### Filter Design and Implementation

To implement A-Weighting in sonometers and other audio signal processors, a common procedure is to include an electronic filter that adjusts the instrument’s measurements. The objective of the filter is to only allow waves inside the human hearing spectrum to go through, applying the intensity modifications that corresponds to their frequency.

In addition to this filter, and in order to obtain more information about the noise spectrum and then, to be able to characterize and classify it, a set of octave filters also have implemented at the embedded system. Most noise measuring devices only provide an absolute value of sound loudness, which is enough to perform noise maps, but not to identify the agents that are generating them. This knowledge could be extremely useful to design and apply noise regulations and their pertinent sanctions, tackling the root motivation of the study: improving overall well-being. In practice, noise level per octave is obtained using a similar procedure to A-Weighting, using electronic filters. In this case, band-pass filters are used, whose central frequencies equal the central frequency of the octave.

In this case, both filters have been implemented as software algorithms, for which coefficient calculations have been developed using MATLAB^®^. First step for obtaining A-Weighting filter coefficients is to call the System object *weightingFilter*, introducing as parameters the weighting method and sample rates desired. This MATLAB^®^ object, extracted from the Audio Toolbox, takes into account non-linearities in the human ear’s sound perception, and has been designed following the IEC 61672-1 standard, used by most professional sonometers in the market. Storing it into a variable, it makes possible to obtain the coefficients to implement the A-Weighting filter as an IIR (Infinite Impulse Response) Filter. This process allows to obtain a code in C that will provide the means for the definition of the A-Weighting filter coefficients towards Keil’s CMSIS DSP Library functions that can be included into the STM32F401RE software development interface.

const float32_t coefficientsAWeighting[NUM_TAPS] = {
0.2343, 0.4686, 0.2343, 1.8939, −0.89516,
1, −2.0001, 1.0001, 1.9946, −0.99462,
1, −1.9999, 0.99986, 0.22456, −0.012607
};

The calculation of octave filters has been developed with a noticeably similar procedure for A-Weighting, described above. As a matter of fact, octave filter coefficients have also been extracted from MATLAB’s system objects, in this case, the ones that can be obtained calling octaveFilter method. This built-in function demands an additional parameter to the one used for the A-Weighting filter, used to specify the central frequency of the considered octave. To produce a MATLAB^®^ method that will be reusable for all the required octaves, this central frequency value will be asked as a parameter when invoking coefficientsOctave, function built during the project to obtaining the octave filter coefficients.

In this case, the coeffs function will return a structure composed of as many elements as the number of stages the IIR filter for the octave has. These elements will be other structures in themselves, that will store two vectors, referencing the denominator and numerator polynomials, respectively. In this context, an additional step for obtaining a unique set of coefficients accepted by CMSIS DSP Library (https://www.keil.com/pack/doc/CMSIS/DSP/html/index.html accessed on 1 March 2021) will be needed. Specifically, convolution operations are required, mathematical procedures that are applied to both the numerator and the denominator of all stages separately, obtaining a unique set of polynomials for each filter stage. These polynomials will be then transformed into an SOS Matrix shape using the tf2sos method. To complete the process, the coefficients in the matrix will be transformed to fit the criteria defined for IIR implementation in C. Next lines present the coefficients created for the 1 kHz octave filter.

const float32_t coefficientsOctave1000Hz[NUM_TAPS] = {
0.04475, −7.1858e-09, −0.044751, 1.8954, −0.91178,
0.04475, 0.089501, 0.04475, 1.9116, −0.94211,
0.04475, −0.089501, 0.04475, 1.9588, −0.96799
 };

## 4. ZARATAMAP Software Architecture and Operation Web Server

The overall objective of the project was to provide public administrations with a set of tools that will enable them to obtain information on noise impact in the city, quantify it, geo-locate it, and characterize it. Through the network of mobile sensors that ZARATAMAP wants to deploy in the city using the bike-sharing service, large volumes of information will be obtained that will provide more accurate information than is currently available. All this data must be sorted, classified, and provided to users through a simple, friendly, and usable interface. This section describes the software architecture created for this purpose, as well as its functionalities.

**Figure 4:**
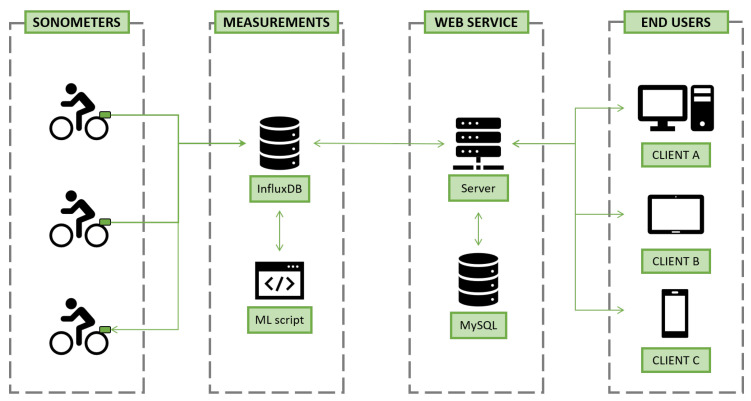
Four-layer-based system architecture.

The software architecture defined for this project is based on the operation of six different types of agents: sonometers, measurement storage database, noise-source identification software, web service provider server, web-service enhancing information database, and end-users’ client devices. These agents can be further classified in a four-layer system architecture, in which multiple direction inter-agent data exchange is supported. This architecture, alongside a high-level vision of the communication between agents, is represented in Figure 4, where gray dashed lines refer to the architecture layers, and icons to the various agents:

- Sonometer: IoT device developed in the complementary Electronics Final Degree Project, around whose support this project has been designed. It is an autonomous noise capturing device that performs the signal’s level measurements, octave-band dissociation, and geo-localization, sending all the data periodically to a central database (InfluxDB database).
- InfluxDB database: Storage system implemented to save the measurements sent by all the intelligent sonometers participating on the program. It is based on InfluxDB, a time series technology focused on performing effective IoT data operations.
- ML script: Custom-made, continuously running computer program that classifies the measurements in the InfluxDB database according to pre-established urban noise origin categories. It is based on a machine learning application that uses the measurements’ octave band dissociation as input to generate a prediction.
- Server: Back-end server supporting the web service upon which this project stands. Developed using Django framework, it handles the communication with the project’s databases and the render of dynamic and interactive web pages, to be returned to querying clients.
- MySQL: Database implemented to store user-related information and data required for the customization of the web service’s maps (specially, for dynamic noise map generation).
- End users: Individuals interacting with the web service, the physical devices through which they perform the actions and the application’s client-side code running in their browsers. This service is targeted to various types of end-users, with different levels of access, analyzed in the following chapters.

The interaction between all the previous actors has been defined according with the following rules:

- Interaction between Sonometers and InfluxDB database: The data retrieved by the sonometer, containing processed-signal and geo-positioning information, are sent to the InfluxDB database by means of an HTTP POST instruction. When retrieving this specific type of message, the database, running in the central control system’s hardware architecture, stores the measurement appropriately.
- Interaction between InfluxDB database and ML Script: The interaction between these two agents, whose objective is to classify the sonometer measurements according to their main noise origin, is done by means of a scheduled program, the ML Script. This script will be continuously running, triggering the execution of the actions displayed on the sequence diagram every day at 00:00 h. At that moment in time, it will query the database to retrieve the undefined measurements; data records that would have been obtained in the last 24 h. For each of the retrieved measurements, the script will predict the noise source using the machine learning model, updating the class of the original data record in the InfluxDB database.
- Interaction between Server, InfluxDB, MySQL, and End-User: These interactions embody the web-service’s functionalities: login, logout, registration, data visualization, data filtering, and report generation.

### 4.1. User Interface Design

One of the key objectives of this project was to provide a publicly available web service, implementing Geographic Information System (GIS) principles to make urban noise measurements accessible for both public workers and the general citizenry. In order to effectively achieve these goals, the system requires the design of intuitive and professional web pages, design that has been materialized by means of User Interfaces. To ensure an all-inclusive GUI design-coherence is achieved, an HTML inheritance approach has been followed. This practice, centered around the idea of having a parent template, from which n children templates are developed, allows to define common HTML, JavaScript, and CSS code for all inheritors, promoting code reusability and visual coherence preservation.

**Figure 5:**
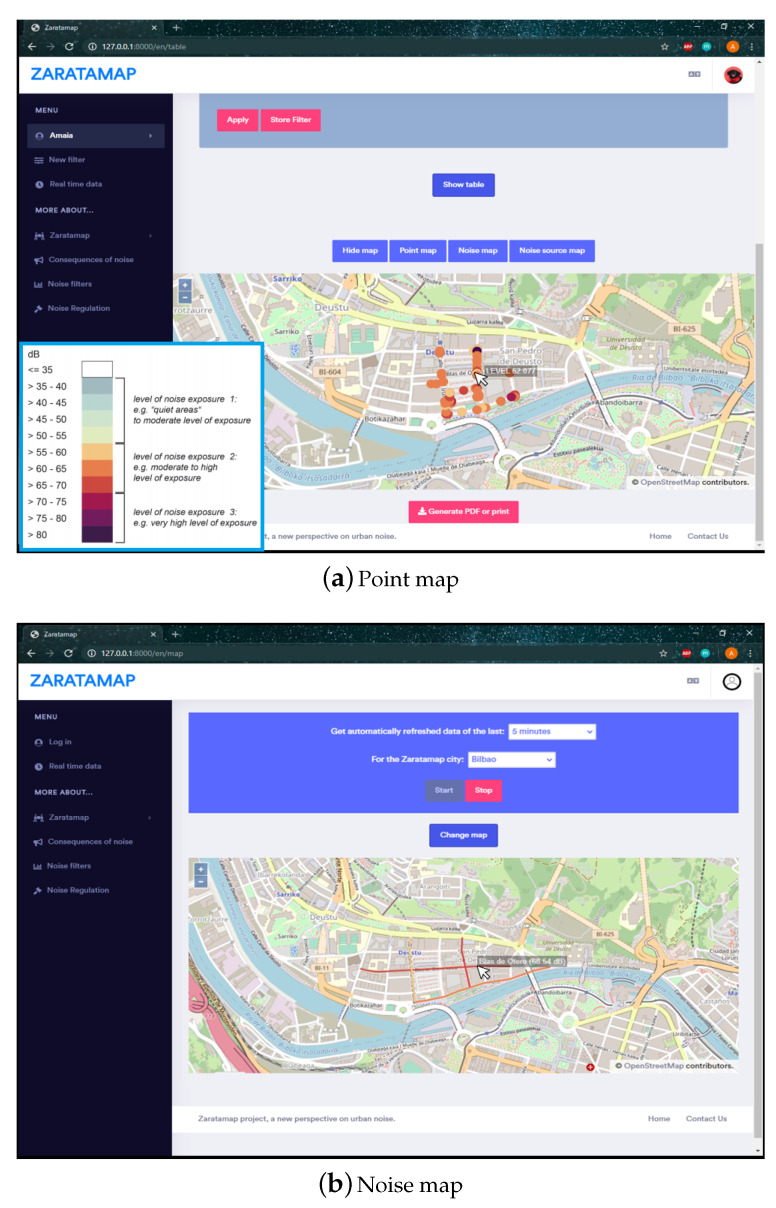
Graphical representation of noise levels in real-time.

The main interface of ZARATAMAP Operation Web Server provides two main functionalities: (a) display information in real-time about the data gathered by the set of mobile sensors spread on the city; and (b)access to data storage in the database. In both cases, the information can be shown with two different formats:

- Point map: The measurements obtained from the database are displayed as individual circular-shape points, colored in relation to their noise-levels (The color code used to represent the noise level has been included in the lower left corner of the figure). When hovering the mouse over one of these points, a small tool-tip indicating its noise level is displayed next to it, as it can be seen in the example gathered in Figure 5a.
- Noise map: The measurements obtained from the database are linked to the Streets they are located in, using their geo-localization fields as base for the spatial calculations. With that data arrangement, each of the affected Streets’ noise level is obtained as the mean of the individual measurements allocated in them. This information is used to paint the concerned Streets on the map, following the same color schema used for the Point map. In addition, similarly to the previous map, hovering the mouse above a colored street visualizes a text containing the street name and its calculated overall noise value, feature represented in Figure 5b.

#### Filter Tool

**Figure 6:**
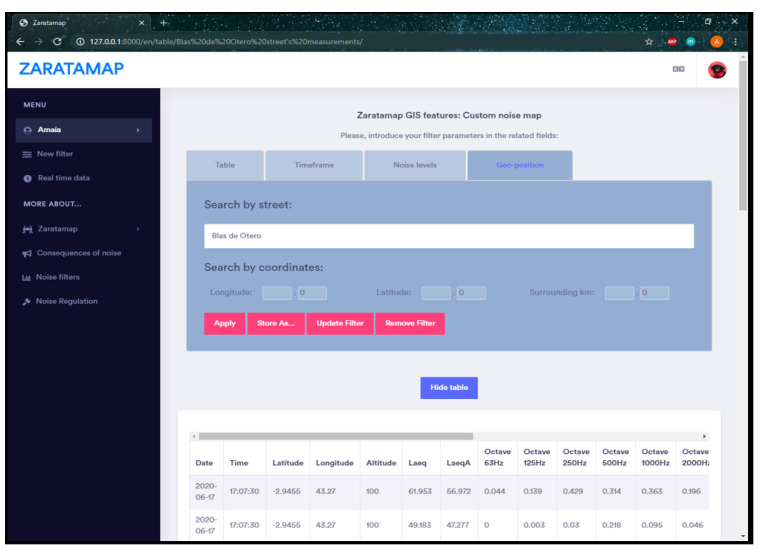
Filter example and table style visualization.

Through the user interface, it is also possible to access the filter generation tool. Through this utility, the user can select different ways to access the information, which will be shown to him in a graphic way or through tables on the screen. These filters are divided into four groups, each one represented by a tab, whose name can be clicked to access its contents. It is important to point out that all filters are applied simultaneously, independently of what tab or position they are located in. These tabs are:

- Table: Filters used to configure the visualization of the measurement displaying table. A unique characteristic of this set of filters, not applicable to the ones in the rest of the tabs, is that they are applied after the data have been retrieved from the server (Figure 6).
- Timeframe: Filters related to date and time ranges between which the measurements were taken. Two types of timeframe filters are considered: range of dates and range of times of the day. If one of the range limits is left blank, the other one is applied as sole upper/lower time limit.
- Noise levels: Filters related to the overall noise of measurements (values in the octaveband dissociation are not considered). This filter group offers two options: to retrieve measurements whose overall noise level fits into a manually introduced range; or to gather those data records whose noise level is higher than the ruled maximum night (50 dB) or day (55 dB) values.
- Geo-position: Filters related to the geographical coordinates in which the measurements were taken. There are two types of filters in this tab: search by street name; and definition of a circular area around a fixed coordinate position (with the specified kilometer radius).

### 4.2. Machine Learning Module: Noise Characterization

The objective of the development of this module was to create an algorithm that is capable of making predictions or decisions, without being explicitly programmed to do so; that instead, it is able to automatically characterize the noise captured by the mobile sonometer and which is sent to the Operation Web Server.

To this end, a machine learning model evaluation–cross validation has been applied. It is an statistical method used to estimate the skill of machine learning models. It is popularly used to compare and select a collection of models, providing lower bias than other methods employed to the same end. Additionally known as rotation estimation, it stands on a resampling procedure, applied during the training of the model by running the learning algorithm and for the evaluation of the accuracy of the learned function.

The basis of cross validation is the partition of sample data into complementary subsets (each data record only belonging to one subset), using one part of the subsets for training the model (training set) and the the other to validate its results (validation or test set). In this context, Cross-validation techniques’ objective is to test the machine learning model’s ability to predict new data, not used to train it, in order to detect overfitting or selection bias problems and give insight on how the algorithm would generalize to an independent (unknown, retrieved from the real environment) dataset.

There are two main types of cross-validation techniques:

- K-fold cross-validation: Type of non-exhaustive cross-validation technique where the original data sample is partitioned into k equal-sized subsets, not following a particular criteria for the arrangement (splitting is performed randomly, and is not affected by classes or groups). Of the k subsamples, one is left aside for validation on the model, using the rest of the k-1 subsets as training data for the model. In this technique, the cross-validation is repeated exactly k times, ensuring all k subsets have been, at exactly one point of the analysis, used as validation data. At the end, it is a common procedure to obtain the single average estimation of the k results. The most popular k-fold cross-validation technique refers to a k equal to 10, option that will be followed during the work presented in this paper.
- Stratified K-fold cross-validation: Variation of K-fold cross-validation where stratification is based on class labels, dividing the sample data in such a way that each set contains approximately the same percentage of samples of each target class as the complete set. It is useful for classification problems where there is a fairly great imbalance in the distribution of target classes, allowing to ensure that every class is equitably represented in each fold. In the case of this project, the way in which the custom dataset has been obtained, can lean towards the benefits of using this method. This is due to the fact that audio samples belonging to different noise-origin classes tend to have different audio lengths, and therefore, produce a significantly different amount of data samples.

Each piece of noise recorded by the mobile sonometer is sent to the InfluxDB database including a unique value for each of the following octaves: 63 Hz, 125 Hz, 250 Hz, 500 Hz, 1000 Hz, 2000 Hz, 4000 Hz, 8000 Hz, and 16,000 Hz. These values correspond to the Root Mean Square (RMS) values of 1 s length audio fragments, calculated after the signal has passed through the corresponding octave filter. The goal of the machine learning application is to use these nine values, floating point numbers in a 0 to 3.3 Volt range, as input to a model that, with those values, is capable of predicting the main source of the noise fragment they belong to. At this point, it is important to remark that everyday noise measurements, especially in urban environments, are not related to a unique origin, but rather to the combination of multiple noises. In fact, these origins could even be considered impractical to be categorized in the way a machine learning algorithm needs, as origins can be very diverse and different levels of detail can be recognized.

#### 4.2.1. Dataset Source

Machine learning model training requires an extensive amount of data to reach reasonable accuracy and reliability. Ideally, these data are obtained through field-operation measurements, ensuring real and representative project data are used to train the model. However, this method of data acquisition is not always feasible, especially when the model is required to be trained and tested before the deployment of the devices whose data are going to be predicted in the first place. This is the case of this project, where the development of the sonometer device and the machine learning model have been carried out in parallel, impeding the retrieval of enough representative data for training. Nevertheless, these circumstances do not put an end to the development of self-learn algorithms. In fact, this problem is fairly common among many machine learning development projects, which has motivated the materialization of an alternative: using pre-categorized datasets, easily purchasable or open-sourced from the Internet.

In this project, UrbanSound8K Dataset has been used. This opensource dataset contains 8732 4 s or less sound excerpts, labeled into 10 urban noise classes: air conditioner (3886 samples), car horn (951), children playing (3923), dog bark (2919), drilling (3377), engine idling (3791), gun shot (444), jackhammer (3224), siren (3609), and street music (4000). Concretely, the downloadable package provides two resources: audio and metadata. At this stage of the project, the noise characterization models only have been trained with these types of noise sources as proofs of concepts. In case a noise was not among them, the model would generate “indeterminate sound” as output. Even so, the models we have worked with are prepared to be trained with other datasets. Thus, in the case of wishing to characterize other noise sources, such as different types of vehicles (trucks, cars, motorbikes, or buses), it would be only necessary to have a sufficient number of samples of these types of noise to train the models.

#### 4.2.2. Machine Learning Model Selection and Model Training

The success and accuracy obtained with a machine learning application is strongly related to an adequate selection of the algorithm, the model, to be used for a problem. There is no learning algorithm that works best on all supervised learning problems, so many considerations must be weighted, including the amount of training data, the dimension of samples, the noise in output values, the heterogeneity, and redundancy in data.

Due to the amount of factors that could potentially impact the success of the choice, a common approach to this step of the process is to perform a comparison of learning algorithms, experimentally obtaining their accuracies to select the one which best performs on the given problem. On that premise, for the development of machine learning capabilities in this project, six popular statistical models have been selected and tested: Logistic Regression (LR), Linear Discriminant Analysis (LDA), K-Nearest Neighbors (KNN), Classification and Regression Tree (CART), Gaussian Naive Bayes (NB), and Support Vector Machine (SVM).

Regarding the model training, the first step is to retrieve the dataset to be used as training and validation data. In this case, the custom dataset is scattered in 10 different files, each of which represents a different fold in the original dataset. In this context, as a first approach for model training, the folds already defined in the UrbanSound 8 K dataset are followed. In order to do so, the individual CSV files have been retrieved using Python Pandas library (https://pandas.pydata.org accessed on 1 March 2021), attaching to each of them the names for the comma-separated fields, so they can be independently accessed in an easy manner. Each individual fold has been stored as a unique dataset in a global array that stores all 10 separately. In case of needing to characterize other types of noise, it would be necessary to re-train the models according to the chosen patterns.

Next, the six models to be analyzed have been declared using Python’s machine learning scikit-learn library. To iterate over the six and analyze their results later in the process, the models have been stored next to their name (in a tuple form) in a sole array. After this step, model training and cross-validation have been performed. Once the model is trained, the accuracy of the fold as a whole and the individual accuracy per class can be obtained and stored in the corresponding array, to be finally transferred to a JSON file at the end of the script. This method returns a structure that can be then used to automatically train and obtain the results of each model.

## 5. Results

This section will analyze the results obtained during the testing phase of the machine learning module developed during the project. In the sections in which both the embedded system and the software architecture in charge of transmitting, storing and representing the information have been described, evidences of their development have been shown.

The selection of the model(s) to be used in the machine learning application has been performed following a comparison of the different models’ accuracies, extracted from the JSON in which they have been stored after obtaining them. Concretely, the overall accuracies for each of the folds in the employed cross-validation technique, as well as the per class accuracies for each of them, have been compared, using a visual representation of data to perform the final decision.

The visual representation has been developed using Python’s (matplotlib (https://matplotlib.org/stable/index.html accessed on 1 March 2021) library. When this script is executed, an addition of a ‘1’ after the name of the file triggers the visualization of the first approach (manual folds), whereas any other execution calling triggers the default visualization of the stratified fold results.

In detail, a 3 × 2 subplot configuration has been used, representing each of the 6 models in its own graph, over which the name of the model has been indicated. In each of the plots, the overall accuracy of each of the folds has been represented using a blue bar. Therefore, each of the graphs displays 10 horizontally distributed bars, one for each fold, following the logical numerical order from left to right (k = 1 on the left and k = 10 on the right).

**Figure 7:**
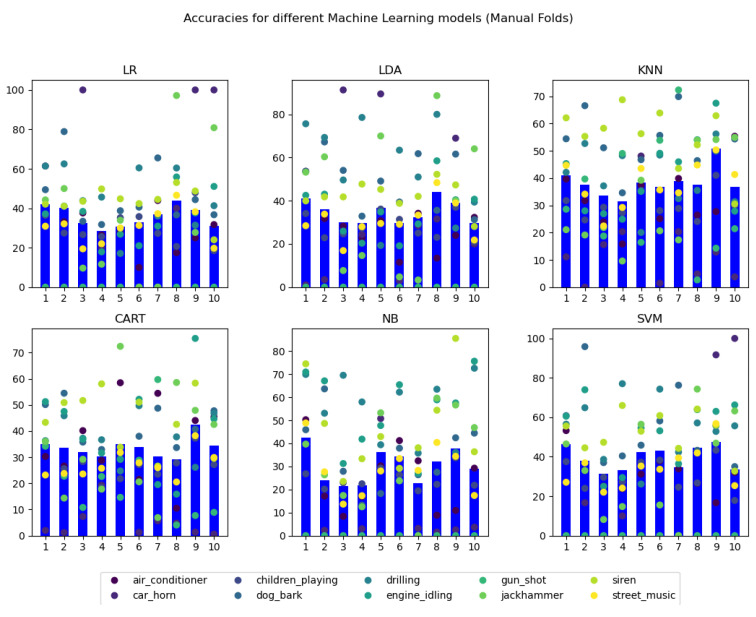
Graphical visualization of the accuracy results for manual k-fold cross-validation.

**Figure 8:**
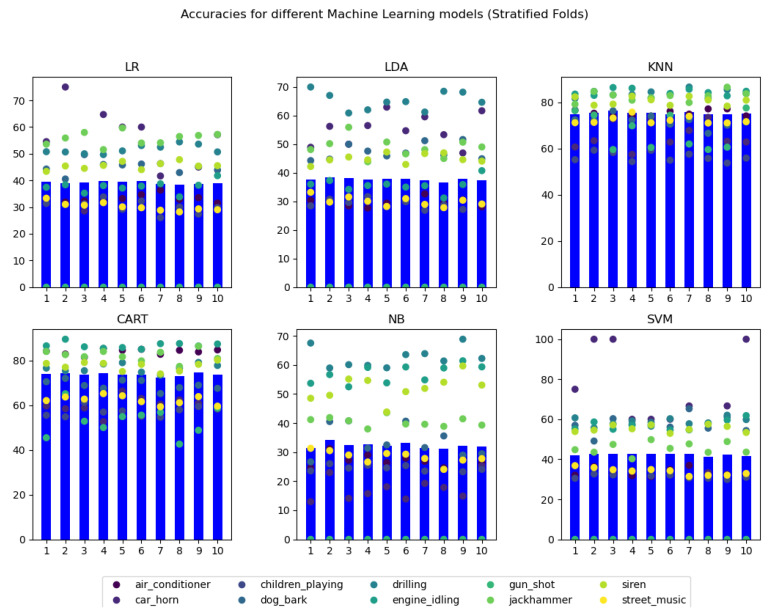
Graphical visualization of the accuracy results for stratified k-fold cross-validation.

Over each of the bars, differently-colored points have been scattered, in such a way that over the whole 10 folds of the 6 graphs, each unique color represents the same class. Under that premise, over each of the model graph bars’, 10 distinctly colored points are represented. The exact correspondence between color and class is indicated in the legend located in the bottom of figures which displays the results. The results obtained for the manual identification of folds (approach one at Figure 7) and the automatic stratified performance of the cross-validation (approach two at Figure 8) are attached in the following figures. In these figures, the Y-axis represents the classification accuracy in the range from 0 to 100%. Then, each blue bar represents the cumulative precision obtained during the classification of all samples analyzed by each of the 10 folds.

From these graphical representations of the accuracies, it can be easily seen that, for all the 6 models, the use of stratified folds produces higher and more uniform results for each of the folds. Per class results are also highly enhanced and uniformed.

At this point, it is remarkable the results obtained for the gun shot class in both manual and stratified folds. It can be noted that in both approaches, Logistic Regression (LR), Linear Discriminant Analysis (LDA), Gaussian Naive Bayes (NB), and Support Vector Machine (SVM) models are not able to obtain a prediction for this class, obtaining a final result of 0 accuracy. A possible cause for this outlier result could be that the number of total samples classified in the used dataset as gun shot is significantly lower than the rest of the classes (it is equivalent to less than a 1% of the total number of samples in the dataset). In fact, this noise origin is related to a very quick sound, whose audio excerpts in the original UrbanSound 8 K dataset are equal or less than 1 s long, producing a unique or none data records in the final dataset.

This consideration can also be applied to the car horn category, that represents around the 3% of the total data, obtaining near 0% accuracies in many KNN, CART, and NB models trained with the manual folds approach. However, the accuracies related to this class category are considerably improved using the Stratified approach to fold extraction, revealing the utility of the strategy.

Regarding the models themselves, it can be seen that using the manual folds approach, the 6 models produce fairly similar results, with overall model accuracies approaching a 35% value. Applying the stratified fold approach however, the overall accuracy of two models is drastically improved: K-Nearest Neighbors (KNN) and Classification and Regression Tree (CART), with mean model accuracies of 75.239% and 73.735%, respectively. In fact, in both models, many of the classes obtain even higher results, with accuracies reaching values higher than 80%.

In conclusion, the two machine learning models that provide the best results for this application’s data, and therefore, have been selected to be employed for its classification problem, are K-Nearest Neighbors (KNN) and Classification and Regression Tree (CART), both trained with stratified K-fold cross-validation.

## 6. Conclusions

The work presented in this article presents the main developments carried out in the context of the ZARATAMAP technological innovation project. Prior to the design and implementation of the described prototype, an important comparative study of the different hardware architectures and technologies used in the field of wireless sensor networks was carried out. An analysis was also carried out of both the regulations and the conducted initiatives in the field of dynamic noise monitoring that have been carried out in Europe.

The main objectives of the developments carried out have been two-fold. Firstly, to verify the versatility of current low-cost and low-consumption hardware platforms for running high added value applications. The chosen platform (STM32F401RE) is based on the high-performance ARM^®^Cortex^®^-M4 32-bit RISC. This is a development platform on which different communications interfaces have been deployed (WiFi and GPS), as well as a low cost, consumption, and high sensitivity acoustic sensor, that has proved its worth in obtaining real-time measurements of the acoustic impact on cities. In addition to integrating these hardware devices, the embedded system has shown to be able to carry out an important work of signal processing, which has not only consisted in obtaining the sound pressure levels (Leq), but also in knowing the detail of the noise contribution for each octave band between 20 Hz and 20 kHz (human audible spectrum). This work of signal processing has been possible thanks to the integration of different technologies that allow to obtain more detail about the noise, which is necessary for its later characterization.

Together with the development of the mobile sensor, a software architecture has been designed and developed focused on the definition of an Operations Web Server. The back-end of this server contains all the necessary functionalities for the storage and processing of the information sent by the mobile sensor, being the front-end through which users can access the different visualization functionalities that the ZARATAMAP tool offers.

The main function of the ZARATAMAP web application is to provide real-time information on the acoustic impact on the city. To do this, it uses the geo-referenced information that each mobile sensor transmits to the server. Each GPS position is associated with 11 noise level values: the noise level in each of the 10 analyzed octave bands and the total noise level (LeqA). The information is provided to the user through a GIS system. In addition, the information system provides different levels of filtering, which allows access to the representation of the acoustic impact either for a specific day, by range of days, or during the hours determined by the user. This allows a level of detail that helps the administrator to make decisions based on knowledge of the city’s noise situation.

However, knowing only the acoustic impact is not always enough. To take action, administrations need more information, such as identifying the nature or source of the noise. For this reason, ZARATAMAP also provides a machine learning module capable of predicting the nature of the noise with an acceptable percentage of accuracy. This will make it possible to determine whether an area of the city is particularly affected by traffic or whether the established municipal regulations regarding the timetables for carrying out work on the infrastructure are being complied with.

The following tasks are envisaged as future work:

- To carry out acoustic calibration of the prototype in a certified environment. During this phase of the project, it has only been possible to check the veracity of the measurements by comparing them with the NL-42 sound level meter, but not in a certified environment such as an anechoic chamber. This process will have to be carried out prior to the possible industrialization of the product by the company participating in the project.
- Improve the accuracy of measurements, mainly with regard to the prediction process. It must be taken into account that in the machine learning system a learning period is necessary, in which the more noise samples available, the more accurate it will be. So while we achieve better results using more octave bands, in particular using octave third-band filters in MatLab, we are analyzing the signal processing and memory capacity of the current embedded system to carry out this processing. These accuracy improvements may be needed for an embedded system with more memory or moving some of the signal processing to the central server, although this could require a redesign of the system.
- Carry out a pilot test in the city of Bilbao, deploying the greatest possible number of mobile sensors. To this end, the aim is to improve the prototype by adding more environmental sensors, so that it can be a more attractive integral solution for the municipality, while also encouraging citizen participation.

With the development of these improvements, as well as an extensive testing and validation process, SAITEC, an enterprise that is part of the project, will have the opportunity to begin the design and development phase of industrialization of the prototype to obtain a commercial product, if after a market analysis it is found to be viable.

## Figures and Tables

**Table 1:** Comparative analysis of available development boards.

Development Board	Arduino Uno	Raspberry Pi 4	FRDM-KL25Z	STM32F401RE
Memory	32 KB (Flash) 2 KB (SRAM)	microSD 1, 2 or 4 GB (SDRAM)	128 KB (Flash) 16 KB (SRAM)	512KB (Flash) 96 KB (SRAM)
Clock	16 MHz	1.5 GHz	48 MHz	Up to 84 MHz
A/D Converter	10 bit 6 channels	Doesn’t have any External ADC is needed	16-bit SAR ADC	1 × 12 bit Up to 16 channels
I/O Ports	14	40	66	81 I/O ports with interrupt capability All 5-V tolerant
Operating Voltage	5 V	5 V	5 V	5 V
Communications	Serial 4 SPI TWI 1 UART USB I2C	USB HDMI TVDAC Bluetooth Gigabit Ethernet	2 8-bit SPI USB 2 I2C 3 UART	3 I2C 3 USART 4 SPI SDIO USB2.0
Dimensions	68.6 × 53.4 mm^2^	88 × 58 × 19.5 mm^3^	81 × 53 mm^2^	82.50 × 70 mm^2^
Price	24 €	64 € (4 GB)	20 €	14 €

**Table 2:** List of commercial acoustic sensors.

Sound Sensor	Grove Sound Sensor	VMA309	IMP34DTO5	Adafruit I2S MEMS Microphone SPH0645LM4H
Operating Voltage	5 V	3.3–5 V	1.6–3.6 V	1.6–3.6 V
Operating Current	4–5 mA	10–15 mA	650 mA	600 uA
Sensitivity	−52–48 dB (1 KHz)	−48–66 dB	−26 dBFS	−42 dB
Frequency Range	16 Hz–20 KHz	50 Hz–20 KHz	100 Hz–10 KHz	50 Hz–15 kHz
S/N Ratio	54 dB		64 dB	65 dB
Dimensions	20 × 20 × 5 mm	44 × 15 × 10 mm	3 × 4 × 1 mm	16.7 × 12.7 × 1.8 mm
Price	5 €	6.34 €	2.1 €	7 €

**Table 3:** Comparison of WiFi modules.

WiFi Module	ESP-01 (ESP8266 SoC)	RTL8710 WiFi Module (RTL8710 SoC)	Air602 (W600 SoC)
CPU	Tensilica LX106 80/160 MHz	ARM Cortex M3 166 MHz	ARM Cortex M3
WiFi 802.11n	Up to 65 Mbps	Up to 150 Mbps	Up to 150 Mbps
Built-in antenna	Yes	Yes	No
Interfaces	UART SPI GPIO I2C	UART SPI GPIO I2C	UART SPI GPIO
Interface AT+ Instruction Set	Yes	Yes	Yes
Operating Voltage	3.3 V	3–3.6 V	3.3 V
Current consumption	80 mA	80 mA	110 mA
SoC chip dimensions	5 × 5 mm	6 × 6 mm	12 × 10 mm
Module dimensions	14.3 × 24.8 mm	24 × 16 mm	12 × 10 mm
Price	<1 €	3.18 €	1.75 €

**Table 4:** Comparison of GPS modules.

GPS Module	BK-SIM808	Ublox NEO-6M	Grove GPS Module
Accuracy	2.5 m	2.5 m	2.5 m
Cold Start	30 s	27 s	13 s
Warm Start	28 s	27 s	1–2 s
Hot Start	1 s	1 s	<1 s
Update Rate	5 Hz	1 Hz, max 5 Hz	1Hz, max 10 Hz
Sensitivity	−165 dB	−161 dB	−160 dB
SIM Card Features	Yes	No	No
Operating Voltage	5 V	3–5 V	3.3–5 V
Module dimensions	37 × 37 mm	23 × 30 mm	40 × 20 mm
Price	14 € (+2 € antenna)	23 €	21 €
